# Mechanism of subclinical hypothyroidism accelerating endothelial dysfunction (Review)

**DOI:** 10.3892/etm.2014.2037

**Published:** 2014-10-27

**Authors:** MING LU, CHONG-BO YANG, LING GAO, JIA-JUN ZHAO

**Affiliations:** 1 Department of Endocrinology and Metabolism, Shandong Provincial Hospital Affiliated to Shandong University, Jinan, Shandong, P.R. China; 2 Institute of Endocrinology and Metabolic Diseases, Shandong Academy of Clinical Medicine, Jinan, Shandong, P.R. China; 3 Scientific Center, Shandong Provincial Hospital Affiliated to Shandong University, Jinan, Shandong, P.R. China

**Keywords:** subclinical hypothyroidism, endothelial dysfunction, lipid profile, oxidative stress, chronic inflammation, thyroid stimulating hormone receptor, L-thyroxine replacement therapy

## Abstract

The association between subclinical hypothyroidism (SH) and cardiovascular disease has received increasing attention in recent years. The predisposition of patients with SH to endothelial dysfunction, an early sign of atherosclerosis, has been observed. This predisposition may be partially explained by the factors also found in patients with SH, including changes in lipid profile, low grade chronic inflammation, oxidative stress and insulin resistance. The proportional risks of endothelial dysfunction to thyroid stimulating hormone (TSH) also indicate that the action of TSH on extra thyroidal-stimulating hormone receptor (TSHR) is a possible mechanism underlying the correlation, which has later been supported by the associated basic studies. L-thyroxine replacement therapy appears to improve the aforementioned aspects, whereas there remain certain controversies, particularly for the elderly. Thus, more study data are required to confirm the benefit of L-thyroxine treatment for patients with SH.

## 1. Introduction

Subclinical hypothyroidism (SH) denotes a declined thyroid activity without clear symptoms, but with elevated thyroid stimulating hormone (TSH) and normal range free thyroxine (FT4) and triiodothyronine (FT3) as the diagnostic indicators. Depending on the extent of serum TSH elevation, SH can be divided into mild (where the concentration of serum TSH is in the range of 4.5–9 mU/l) or severe (TSH≥10 mU/l) SH (1). Mild SH constitutes ~75% of the total number of patients with SH. SH affects 4–20% of the adult population, influenced by factors such as age, gender, race, body mass index, dietary iodine intake and the inconsistency of the boundary point of serum TSH for SH diagnosis amongst the studies (2). In patients aged >60 years, the diagnosis poses a challenge as thyroid function test results may be affected by certain physiological changes due to no thyroidal illnesses following ageing (3).

Evidence supporting the correlation between SH and atherosclerosis has been accumulating. The first study regarding the associated cardiovascular risk in patients with SH was the long-time large cross-sectional Rotterdam study in the Netherlands, which showed an increased risk for atherosclerosis and prevalence of myocardial infarction among female patients with SH aged >55 years (4). Subsequently, another study also observed an increased risk of congestive heart failure among older patients with SH that had a TSH level >7.0 mU/l, but no significant correlation between SH and stroke, peripheral arterial disease, cardiovascular-associated or total mortality was revealed (5). Another cross-sectional analysis further revealed SH to be an independent risk factor for coronary heart disease, in parallel to hypercholesterolemia, hypertension, smoking and diabetes (6). The association between SH and ischemic heart disease and the associated mortality was also confirmed by the unselected community-based, 20-year follow-up the Whickham survey (7). In previous years several meta-analyses have supported these conclusions from the perspective of evidence-based medicine. One significant meta-analysis based on the previous studies from 11 countries, including in the Americas, Europe and Asia, showed that SH is associated with the coronary heart disease incidence and mortality, particularly for the group of TSH≥10 mU/l (8). A meta-analysis from Taiwan also supported this conclusion (9). Another study identified the original cohort studies with a systematic review and obtained a more precise estimate of the risks of the cardiovascular outcomes associated with SH (10). Since the association between SH and cardiovascular disease is becoming increasingly convincing, it is intriguing to understand the mechanisms for the association.

Atherosclerosis is characterized by extensive porridge-like lipid deposit plaques in the great arterial wall, and accounts for a vast majority of cardiovascular disease incidence. Therefore, this association is the first to be assessed for the link between SH and cardiovascular disease. Although a detailed pathogenesis remains to be studied, atherosclerosis is believed to be initiated by a combination of special shear flow condition, sub-intimal lipoprotein deposition and modification, and endothelial dysfunction (11). Progression of atherosclerosis features a chronic unresolved inflammatory response, leading to fibrosis, tissue necrosis and thrombosis (12). Since atherosclerosis is a chronic disease, to observe the whole pathogenesis in one single investigation would require a long time of study (13). Thus, endothelial dysfunction, as one of the earliest signs for atherosclerosis, could be most frequently observed in clinical investigations (particularly prospective ones), prior to any overt manifestations of cardiovascular disease (14). Therefore, endothelial dysfunction would be a favorable initial factor to investigate the correlation between SH and cardiovascular disease.

Endothelium dysfunction contributes to atherosclerosis in a number of ways. In normal conditions, vessel smooth muscle cells are dependent on a variety of vasoactive agents released by endothelial cells, including nitric oxide (NO), thromboxane (TXA_2_) and prostacyclin (PGI_2_) to dilate and constrict efficiently (15). Among these substances, NO, synthesized by endothelial NO synthase (eNOS), has been recognized as one of the most important vasodilating agents, since an array of vasodilating substances have been found to be dependent on NO to exert their function (16).

eNOS is constitutively expressed in endothelial cells, however, inducible NOS (iNOS) is more powerful in NO synthesis but is induced only by inflammatory stimuli (17). NO acts as a second messenger activating guanylate cyclase in smooth muscle cells, which can lead to protein kinase G activation leading to smooth muscle cell hyperpolarization and subsequent relaxation, but the mechanism that is independent of guanylate cyclase has also discovered (18). As NO has a short half-life, which is approximately seconds, vascular smooth muscle cells require a constant source of NO to maintain a normal function. During increased shear stress condition, the change of blood velocity can induce NO synthesis and lead to quickly adapted vasodilation, which is the principle of mediating flow-mediated dilation (FMD) as an indicator for endothelial function. Declined NO activity caused by various factors impairs vasodilation in response to various stimuli, accelerates recruitment of macrophages into the vascular wall, promotes platelet adhesion, aggregation and thrombosis (19). Besides vasodilation, NO also regulates a number of diverse biological processes, such as vascular permeability, neurotransmission, platelet adhesion and mitochondrial respiration (20). Under physiological conditions, the reduction in endothelial NO bioactivity has been taken as a signature for endothelial dysfunction. Using NO activity as the marker of endothelial function can also be justified by another aspect of endothelial function, which is the material exchange between blood and tissue. Endothelial NOS function is also found to be regulated by caveolin-1, the main component of the endocytosis structure caveolae located in endothelium surface (21), which also mediates low density lipoprotein (LDL) endocytosis contributing to atherosclerosis (22). Therefore, NO activity appears to be able to integrate various aspects of endothelial function, and thus could be taken as a good indicator.

In clinical practice, endothelial dysfunction could be represented by endothelium-dependent vasodilation dysfunction, which reflects, in large part, the action of endothelial-derived vasodilators, mainly NO. One of the most commonly used non-invasive examinations of endothelium-dependent vasodilation dysfunction is using vascular ultrasound to measure the FMD of the conduit brachial artery. Other non-invasive methods to monitor arterial stiffness as an indicator for endothelial function are also available, including pulse wave velocity and arterial distensibility measurement (23). Carotid intima-media thickness (CIMT), which indicates the extent of lipid deposition, is also a common marker of early atherosclerosis.

Previously, microRNAs (miRNAs or miRs), a class of short, single-stranded, small non-coding RNAs regulating gene expression, emerged as a novel aspect in several diseases, including endothelial dysfunction (24). Since miRNAs are highly expressed in endothelial cells, circulation miRNA is mainly composed of endothelial miRNA. Serum miRNA may be an extremely promising indicator for endothelial function. The significance of different miRNAs concerning endothelial function is undergoing increasing investigations. For example, while miR-10a and miR18b appear to suppress nuclear factor-κB (NF-κB) downstream signaling by inhibiting NF-κB nuclear translocation, miR-146 has been shown to promote NF-κB activation and eNOS expression. Shear stress, one of the factors affecting endothelial dysfunction, has been found to induce miR-10a and miR-92a (25). miR-92a is also upregulated by oxLDL, which promotes endothelial activation and the development of atherosclerotic lesions (26). There are also several miRNAs that can reflect the change of endothelial function in atherosclerosis, such as miR-34, miR-217, miR-146, miR-126, miR-92a and miR-21, and these have been reviewed in a previous study (27). In addition to the classical biomarkers, such as C-reactive protein (CRP), miRNAs may be translated into novel therapeutic approaches and even be available as approved drugs to humans in the future. As the association between different miRNAs and atherosclerosis is becoming clear, using miRNAs to understand the endothelial change in different conditions would be feasible.

For example, the present study is trying to establish novel indicators linking SH and endothelial function. Our previous study (data not published) indicates that several miRNAs, including miR-125a and miR-21-5p, all change in SH. The results will be published in a forthcoming issue (28).

## 2. SH accelerates endothelial dysfunction

Patients with SH have been found to be disposed to endothelial dysfunction. In one clinical study, Turemen *et al* (29) used brachial artery responses to endothelium-dependent (FMD) and endothelium-independent stimuli [sublingual nitroglycerin (NTG)] as indicators for endothelial dysfunction, and found that when confounding factors were excluded, patients with SH have a statistically lower FMD and NTG response compared to the controls, with FMD impairment correlating to serum TSH level. This study not only revealed the increased endothelial dysfunction incidence in patients with SH, but also indicated a possible role of serum TSH in this phenomenon. Subsequently, the direct effect of TSH on vessel dilation was observed in the study by Dardano *et al* (30), which discovered impaired FMD following recombinant human TSH (rhTSH) acute administration in patients monitored for differentiated thyroid carcinoma. In this study, the inflammation and oxidative stress indicators were also assessed and implied as a possible interpretation for the link between TSH and endothelial change.

In the past 10 years, several studies have indicated that SH is associated with increased CIMT, but the data are sometimes inconsistent. Recently a meta-analysis that included eight observational studies with 3,602 SH patients fulfilling the eligibility criteria made a conclusion that SH was associated with an increase of CIMT correlated with TSH elevation, particularly when TSH >10.0 mU/l. An increase of IMT was also found in patients with TSH <10 mU/l, regardless of significant heterogeneity (31).

The mechanism underlying the correlation remains unknown. In SH, the only noticeable change is the elevation of TSH, and it is possible that the elevation of TSH can bind extra TSH receptor (TSHR) to exert its function. The discovery by Balzan *et al* (32) that TSHR is expressed by microvascular endothelial cells opens up a novel prospective for understanding the association. The function of the TSHR is further confirmed in a recently published study carried out by Tian *et al* (33), which indicates that elevated TSH can promote endothelial dysfunction in human umbilical vein endothelial cells by attenuating eNOS and prostacylin (PGI2) expression in a dose- and time-dependent manner.

Recently, the association between cav-1 and thyroid function has gained increasing attention. One study carried out by Wang *et al* (34) found an upregulation of caveolin-1 in the hippocampus and cerebella of developmental hypothyroidism rat created by iodine deficient diet or propylthiouracil (PTU) administration (35,35) indicating an effect of hypothyroidism on cav-1 function. Another study using adult hypothyroidism and age-matched euthyroid rats not only confirmed the inductive effect of hypothyroidism on cav-1, but also observed decreased eNOS activities and further revealed a possible mechanism underlying the link between hypothyroid and endothelial dysfunction (36). However, since the above findings are all obtained from hypothyroid animal models, whether or not SH has a similar effect on cav-1 requires investigation. However, as TSHRs are located and regulated by constitutive multimerization within lipid micro-domains on the plasma membrane (37), cav-1 and TSHR signaling may be closely associated. As mentioned, TSH can regulate endothelial function directly and therefore, the interaction between cav-1 and TSH may be another significant element to uncover the mechanism of endothelial dysfunction in SH state.

The findings above support a direct effect of TSH during the process. However, the body is an entity and therefore there may be other noteworthy factors that could also impair endothelial function in SH.

## 3. Mechanism of SH accelerating endothelial dysfunction

### Dyslipidemia

Hyperlipidemia is one of the common causal factors of endothelial dysfunction. In endothelial cells, hyperlipidemia can disturb the NO synthesis pathway by increasing levels of asymmetric dimethylarginine (ADMA), the endogenous NO synthesis inhibitor, possibly by reducing enzyme dimethylarginine dimethylaminohydrolase (DDAH) activity (38). High density lipoprotein (HDL) cholesterol has also been reported to improve the endothelial dysfunction by stimulating NO release and inducing vasodilation in the isolated aorta via Akt-mediated eNOS phosphorylation and intracellular Ca^2+^ mobilization, and therefore is protective to endothelial function (39). One of the major components of plaques is lipid, elevation of serum LDL levels is recognized to promote subintimal lipid deposition, and therefore more subintimal modified LDL to aggravate endothelial dysfunction.

However, SH is also known for its correlation with dyslipidemia. Therefore, SH may induce endothelial dysfunction by increased lipid disorders. A study in the DaDong district of Shenyang (China), showed that elevation of TSH, across the entire TSH reference range, exhibits a positive correlation with serum total cholesterol (TC), triglycerides, LDL cholesterol (LDL-C), and a negative correlation to HDL cholesterol. Serum TSH was also found to be positively correlated with the prevalence of obesity, which also suggested that serum TSH may be a risk factor for metabolic syndrome (40). Another cross-sectional, population-based study further showed that subsequent to excluding the possible interference of insulin sensitivity, raised serum TSH remains a risk factor of dyslipidemia (41). Data of the present authors also supports a correlation between SH and atherosclerosis lipid profile changes. The association between elevated TSH and atherogenic lipid profiles (increase of TC, LDL-C and ox-LDL) is observed even with a mild elevation of serum TSH, particularly in postmenopausal females, a population with an increased risk of atherosclerosis (42).

Recently, a clinical study performed by Xiang *et al* (43) showed a close association between increased postprandial lipaemia (PPL) and impaired endothelial function in patients with SH by analyzing FMD change prior and subsequent to an oral fat-loading in overt hypothyroidism, SH and normal control groups. Oral fat-challenged FMD was found to be impaired, while TC, LDL-C, CRP and thiobarbituric acid reactive substance (TBARS) levels were also found to be higher in patients with SH compared to the control group, further indicating a role of dyslipidemia underlying the bias to endothelial dysfunction in patients with SH.

The mechanism between SH and hyperlipidemia has been interpreted to a certain extent. The present authors demonstrated that TSH, acting on the TSHR in liver cells, upregulated the expression of hepatic 3-hydroxy-3-methyl-glutaryl coenzyme A reductase (HMGCR), a rate-limiting enzyme in cholesterol synthesis in the liver. The results revealed the direct effect of TSH on cholesterol levels in the liver form a novel perspective and possibly partially explained hypercholesterolemia in SH (44).

### Low grade chronic inflammation factors

Chronic inflammation may initiate and promote atherosclerosis or its complications by adverse effects on the vascular endothelium, and it may be one of the contributing factors that lead to the increased endothelial dysfunction in patients with SH. The clinical study carried out by Turemen *et al* (29) observed not only an elevation of several inflammation indicators, including interleukin-6 (IL-6), tumor necrosis factor-α (TNF-α) and CRP, in patients with SH, but also a positive correlation of FMD between these inflammation factors, indicating that low grade chronic inflammation may be one of the factors that can promote endothelial dysfunction in SH.

Another study by Taddei *et al* (45) revealed higher CRP and IL-6 values and reduced vasodilation to Ach in the SH group compared to the controls. The reduced vasodilation was resistant to N-mono methyl arginine (L-NMMA), a NOS inhibitor, and normalized by vitamin C, confirming an impaired NO availability. It was also normalized following systemic but not local administration of indomethacin or celecoxib, a selective COX-2 inhibitor, and therefore a COX-2-dependent pathway may be involved. As COX-2 is the induced type of COX that is upregulated during the inflammatory response, inflammation is considered to play a role in endothelial dysfunction.

Various inflammatory mediators, including IL-6, TNF-α and CRP, have been found to be linked to SH, and thus require more study. For example, IL-6, a pro-inflammatory cytokine that is found to be detrimental to endothelium and atherosclerosis (46) is also found to be induced by TSH in preadipocytes (47). TNF-α, another inflammatory cytokine that can impair NO activity in endothelial cells by promoting oxidative stress (another aspect of endothelial dysfunction) (48), has been found to be induced in bone marrow cells by TSH (49). Furthermore, CRP, one of the ‘acute phase proteins’ generated by liver under inflammatory challenge, a traditionally used inflammatory marker later discovered to be indicative of cardiovascular events, is observed to be increased in patients with SH (50), male patients with SH aged <50 years (51), or found to be in positive correlation to TSH (52). CRP can interfere with endothelial function directly by downregulation of eNOS and upregulation of endothelin-1 (ET-1), which is a potent vasoconstrictor that can antagonize NO action (53).

### Oxidant stress

Oxidant stress indicates an imbalance between the oxidant and antioxidant substance, during which reactive oxygen species (ROS), a family of molecules including hydroxyl radical, superoxide anion and their derivatives, exceeds endogenous antioxidant defense mechanisms. Inflammation is an important cause of oxidative stress as enzymatic systems producing a large amount of ROS, including xanthine oxidase and nicotinamide adenine dinucleotide (NADH)/NADH phosphate oxidase (NOX), are induced by inflammatory stimuli. Since ROS reacts with NO extremely readily, producing even more harmful reactive nitric intermediates, minimum oxidative stress in endothelial cells can uncouple NO synthesis and will be devastating to endothelial function (54). Induction of iNOS can therefore aggravate oxidative stress in inflammatory conditions (55). ROS can also impair endothelial function by activating NF-κB, which further increases the expression of inflammation-associated genes (56), and thus act as a negative feedback loop. As inflammation appears to link to SH, it is reasonable to expect another link between oxidative stress and SH.

However, this link was not observed initially. In the clinical study carried out by Coria *et al* (57), several serum indicators of oxidative stress were utilized, including NO concentration, TBARS (a by-product of lipid peroxidation) and paraoxonase (PON, a major component of HDL), to represent the oxidative stress of each individual, and denied a clear oxidative stress in the SH group. However, this conclusion was inconsistent with the studies that followed. Another study by Torun *et al* (58) found an altered level of malondialdehyde (MDA; an indicator for lipid peroxidation) and total antioxidant status (TAS, an indicator for overall antioxidative activity) in SH and overt hypothyroidism (OHT) states compared to normal control. Cebeci *et al* (59) also found lower activity of PON and arylesterase (ARE) in patients with SH. These two studies are supportive of an increased oxidative stress in patients with SH. In the study carried out by Cebeci *et al* (59), while MDA, diene conjugate (DC), protein carbonyl (PC), nitrotyrosine (NT) levels and ferric reducing antioxidant power (FRAP) were all increased in overt hypothyroid patients, only MDA levels were statistically increased in patients with SH (60).

The different conclusions drawn from different biomarkers may partially explain the inconsistencies of the above studies, and also suggest an investigation of how these biomarkers are different and which of them would represent a particular process or stage of oxidative stress, and which would be the most specific to be used as an indicator for body overall oxidation condition. As a correlation between serum TSH and those oxidative stress indicators was also observed (60), a direct effect of TSH to promote oxidative stress is possible. This possibility is supported by the study of Dardano *et al* (30), which found that rhTSH acute injection can lead to oxidative stress. The contribution of oxidative stress to the impaired endothelial function in patients with SH is confirmed in a study (61), which observed a reduction of TBARS levels and a marked improvement of FMD after three weeks of α-lipoic acid antioxidant therapy, indicating a causal association between oxidative stress and endothelial dysfunction in an SH condition. In conclusion, there is growing evidence supportive to a higher overall oxidative burden among patients with SH associated to their impaired endothelial function.

### Insulin resistance (IR)

IR is defined as decreased sensitivity and/or responsiveness to metabolic actions of insulin. IR is associated with dyslipidemia, chronic inflammation and oxidative stress, as facets of metabolic syndrome. As discussed above, dyslipidemia, chronic inflammation and oxidative stress can all directly promote endothelial dysfunction. Metabolic syndrome has been taken as an important underlying cause for the majority of the cardiovascular diseases. The effect of IR on endothelial dysfunction was confirmed by the observation that endothelium-dependent coronary vasodilation is in association with the severity of IR in non-diabetic patients (62). Despite the link with metabolic syndromes, however, IR could also influence endothelial function directly. Insulin stimulates the production of NO and suppresses secretion of ET-1 in endothelium through a phosphatidylinositol 3-kinase-dependent (PI-3K) pathway, leading to vasodilation. IR can therefore lead to the imbalance between NO and ET-1, manifested as endothelial dysfunction, which in turns leads to decreased blood flow, and can worsen IR (63).

The evidence of a predisposition of IR among patients with SH are accumulating. Patients with SH have been found to have a higher homeostasis model assessment of IR (HOMA-IR) score correlated with TSH level (64), higher plasma insulin and increased HOMA-IR score (65), or a higher insulin level only without affecting the HOMA-IR statistically (66). They are also supportive to a higher cardiovascular risk in patients with SH, and the inconsistencies between studies may possibly be due to the difference of insulin level and HOMA-IR in their specificity and sensitivity.

Although it could be attributed to their link to metabolic syndrome, the detailed mechanism underlying the association between SH and IR remains uncertain. Peeters *et al* (67) examined two common polymorphisms of *Tshr*, *Tshr-Pro52Thr* and *Tshr-Asp727Glu*, and concluded that *Asp727Glu* was associated with IR in healthy elderly males. Taking this finding together with the increased IR risk in SH, which is characterized by elevated serum TSH, it is possible that individuals with *Tshr-Asp727Glu* can express TSHRs with a higher affinity to TSH, which can also result in increased TSHR signaling mimicking the SH situation, and thus are predisposed to IR.

## 4. Levo-thyroxine (L-T4) replacement

Levo-thyroxine (L-T4) is the replacement therapy drug used in hypothyroidism. Taking the proper amount of L-T4 aids to increase serum FT4 and FT3 levels in SH, and can reduce TSH secretion with negative feedback on the pituitary. The effect of L-T4 replacement would be useful to verify the causal association between endothelial dysfunction and SH. However, current evidence remains insufficient to confirm the beneficial effect of L-T4 therapy on patients with SH.

In the study carried out by Taddei *et al* (68), after six months of L-T4 replacement, a clear improvement in acetylcholine-induced vasodilatation, associated with a restoration of the inhibitory activity of L-NMMA, was observed. The study concluded that the alteration of lipid profile, inflammatory status and the direct effect of thyroid hormone in patients with SH jointly contributed to their impaired endothelial dependent vasodilation and early L-T4 replacement therapy may be advisable to slow down atherogenesis. Similarly, a double-blind, placebo-controlled L-T4 replacement study, with 45 patients with SH and 32 controls aged <55 years, also observed a significant improvement in lipoprotein profile and IMT after six months of treatment (69).

Another similar clinical study also observed an increased nitroglycerin-induced diameter (NID) following LT4 treatment in patients with SH, although the difference of NID values between SH and the control group prior to the treatment was not statistically significant (70). A randomized, double-blind, 12 weeks crossover study by Razvi *et al* (71) also showed that L-T4 treatment can reduce TC, LDL-C, waist-to-hip ratio and improve FMD in patients with SH, which are all protective of cardiovascular events. The study also noted that, although remaining within the normal range, an increased level of free T4 concentration was associated to the cardiovascular risk factor reduction (71). Another study by Adrees *et al* (72), also reported an improvement endothelial function characterized by an increase of carotid artery baseline diameter, a decreased CIMT and an increased endothelium-dependent vasodilatation. All these investigations indicate that thyroid hormone replacement therapy may be beneficial to endothelial dysfunction in patients with SH.

However, there were also contradictory opinions. Another replacement study showed that although there was a significant decrease in FMD in the SH compared to control group prior to treatment, the improvement of 12 months L-T4 treatment on FMD and mean CIMT were not statistically significant (73). Another study analyzing the United Kingdom General Practitioner Research Database suggested that treatment of SH with L-T4 was associated with fewer ischemic heart disease events in younger individuals, but this was not evident in older people. This finding is consistent with a recent review conducted by Pasqualetti *et al* (74), concluding that the increased cardiovascular risk in SH participants is more evident among the young patients, and the oldest subjects (>85 years) should avoid hormonal treatment due to negative effects of possible overtreatment. An appropriately powered randomized controlled trial of L-T4 in SH examining vascular outcomes is warranted, particularly to value the effect for the older patients (75).

## 5. Conclusion

**Figure 1:**
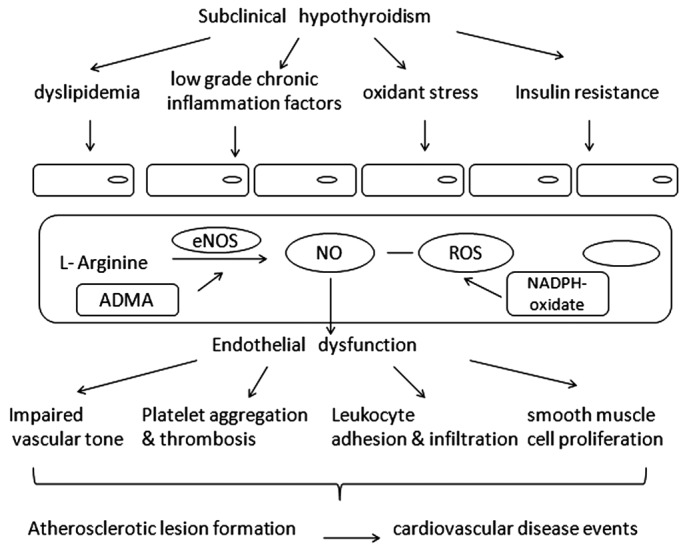
Subclinical hypothyroidism accelerates endothelial dysfunction through four aspects mentioned in the present review. These aspects affect specific molecular pathways in endothelial cells and cause elevated levels of NO and other molecular changes, characteristic of endothelial dysfunction, resulting in atherosclerosis and cardiovascular events. ROS, reactive oxygen species; NO, nitric oxide; NADPH, nicotinamide adenine dinucleotide phosphate; eNOS, endothelial NO synthase.

SH, associated with elevation of TSH while FT3 and FT4 remain normal, is convincingly associated with increased cardiovascular risk. As the earliest sign of atherosclerosis, endothelial dysfunction is most frequently observed in clinical studies. Weakened endothelium dependent vasodilation, together with other indicators of endothelial dysfunction, have been found to be associated with SH, supporting the correlation between SH and cardiovascular disease. The emergence of miRNAs as an indicator for endothelial function may help to further reveal this correlation. From various clinical investigations, factors contributive to endothelial dysfunction, dyslipidemia, chronic inflammation, oxidative stress and IR also link with SH, which partly accounts for the correlation (Fig. 1). However, all these factors interacted with each other, with none playing the decisive role alone. For example, IR is induced by inflammation and oxidative stress, and oxidative stress also plays a role in dyslipidemia. Inflammation and oxidative stress promote each other. All the aforementioned induce endothelial dysfunction, resulting in early stage atherogenesis.

Elevated TSH alone, independent of FT3 and FT4, has been shown to modulate the whole process. It has been discovered that TSH is able to bind hepatocyte TSHR to promote cholesterol synthesis, bind adipocyte TSHR to induce IL-6 synthesis and bind bone marrow cell TSHR to increase TNF-α secretion. A polymorphism in *Tshr* has also been identified to be associated with IR, indicating a possible role of TSH signaling in IR pathogenesis. These actions of TSH are closely associated with altered endothelial function, and therefore could be promising mechanisms underlying the correlation between SH and endothelial function. However, endothelial cells also express TSHR, therefore TSH could also bind to endothelial TSHR to exert its effect, which remains a largely limited area until recently. The study of the contribution of endothelial TSHR to endothelial dysfunction would be noteworthy. Studies on extra thyroidal TSHR function have been supportive to clinical findings, and have complemented the understanding of the observed correlation. The novel association between cav-1 and thyroid function also suggests cav-1 may be another notable element of the underlying mechanism.

Using the currently available data, L-T4 replacement therapy is beneficial for the recovery of endothelial cells from early stage injury in the majority of cases, particularly in the severe SH (TSH≥10 mU/l) group. Early intervention can slow down the progress of atherosclerosis and improve the condition that long-term SH increases the risk of cardiovascular events. However, L-T4 replacement therapy would also increase the risk of osteopenia and atrial fibrillation, and there remain controversial opinions on substitution treatment, particularly for the elderly (76). Therefore, more large-scale and long-term evidence-based medical study data to further demonstrate the feasibility of replacement therapy are required for a finalized conclusion.
